# Longitudinal Effects of Distress and Its Management During COVID-19 Lockdown in Spain

**DOI:** 10.3389/fpsyg.2021.772040

**Published:** 2021-12-22

**Authors:** Patricia Flor-Arasil, Jesús F. Rosel, Emilio Ferrer, Alfonso Barrós-Loscertales, Francisco H. Machancoses

**Affiliations:** ^1^Faculty of Health Sciences, Valencian International University, Valencia, Spain; ^2^Department of Developmental, Educational and Social Psychology, Jaume I University, Castellón de la Plana, Spain; ^3^Department of Psychology, University of California, Davis, Davis, CA, United States; ^4^Neuropsychology and Functional Neuroimaging Group, Department of Basic and Clinical Psychology and Psychobiology, Jaume I University, Castellón de la Plana, Spain; ^5^Predepartamental Unit of Medicine, Jaume I University, Castellón de la Plana, Spain

**Keywords:** longitudinal, distress, gender, lockdown, pooled time series, intensive methods

## Abstract

**Introduction:** The COVID-19 pandemic that hit Spain during March 2020 forced the strict confinement of the population for 2 months. The objectives of this study were (a) to assess the magnitude and duration of the influence of confinement on people’s Distress, (b) to study the temporal sequence of stress, and (c) to show how different day-to-day activities and personal variables influence perceived Distress levels.

**Method:** A daily registration was completed by 123 people, with ages ranging from 21 to 75 years old ($\bar{X}$ = 43, *SD* = 10 years), of which there were 40 men (32%) and 83 females (68%). During 45 days of lockdown, from March 19th to May 3rd, participants were asked to respond to a socio-demographic survey and make daily records comprising the MASQ-D30 and some day-to-day behaviors. Pooled time series was applied to establish what effect time had on the dependent variable.

**Results:** Distress has a 14-day autoregressive function and gender, physical activity, sexual activity, listening to music, and teleworking also influence Distress. It has been hypothesized that the intercept presents variability at level 2 (individual), but it has not been significant. Interactions between Gender—Telecommuting, and Gender—Physical Activity were observed. Approximately 66% of the variance of Distress was explained (*R*^2^ = 0.663).

**Discussion:** At the beginning of the lockdown, the average levels of Distress were well above the levels of the end (*z* = 3.301). The individuals in the sample have followed a very similar process in the development of Distress. During the lockdown, the “memory” of Distress was 2 weeks. Our results indicate that levels of Distress depend on activities during lockdown. Interactions exist between gender and some behavioral variables that barely influence Distress in men but decrease Distress in women. The importance of routine maintenance and gender differences must be considered to propose future interventions during confinement.

## Introduction

The global pandemic situation due to SARS-CoV-2 (COVID-19) made several countries implement exceptional and severe measures to prevent the spread of the illness. The last two decades have seen a growing trend of different epidemic outbreaks that have taken place around the world, such as the polio epidemic in Uttar Pradesh (India), the SARS epidemic, the H_1_N_1_ flu pandemic threat, the Middle East respiratory syndrome coronavirus (MERS-CoV), and the Zika epidemic in Latin America. However, none of these sanitary alerts impacted at the level the COVID-19 has. Not since the 1918–1920 flu pandemic has there been a need to fight against such a high infection rate, and with so many psychological and physical health consequences. During the COVID-19 crisis, worldwide lockdown and quarantine measures were taken, and previous lockdown situations have taken place as happened in several areas of China (Guan et al., 2003) and Canada due to SARS (Hawryluck et al., 2004), and in other African countries during the Ebola virus epidemic in 2014 (Frieden et al., 2014). Hawryluck et al. (2004) report that post-traumatic stress disorder and depression were found in 30% of their studied sample, and that these diagnoses were related to the lockdown period.

In this regard, Brooks et al. (2020) have elaborated a systematic review about psychological impact in quarantine and lockdown, in order to explore the possible mental and psychological wellbeing effects that can be caused, and the factors that may prevent these effects. This revision points out stressors that can account during lockdown (such as duration, fear of infection, boredom, frustration, insufficient resources, and inadequate information), and after isolation due to lockdown (such as economic problems and social stigma). Regarding the effects during lockdown, it has been observed that physical activity seems to work as a protector factor in the prevention of depression, stress, and anxiety symptoms (Stanton et al., 2020). Reducing physical activity during lockdown had a negative impact on mood, as did the decrease in the sleep quality (Chouchou et al., 2020; Ingram et al., 2020). Although it is yet unclear if sleep impairment produces a negative mood or vice versa, several studies have been carried out in relation to it. Anyway, lockdown seems to have changed sleeping patterns (Gupta et al., 2020), following a general tendency of going to sleep and waking up later but perceiving a lower quality of sleep (Cellini et al., 2020).

It has been previously observed that quarantine can lead to emotional and psychological problems (Palinkas et al., 2004), and in order for lockdowns to be effective, not only people at risk should obey it, but also people who are used to having full rights and liberties. These exceptional lockdown situations have been studied in exceptional cases such as astronauts, scientific expeditions to Antarctica, or any other situation where people have been previously trained, generally focusing on the possible causes for tension and interpersonal conflict, or exposure to extreme conditions (Palinkas et al., 2000; Kanas et al., 2001; Rosnet et al., 2004; Steel, 2005; Sandal et al., 2006). No previous studies have investigated the psychological effects on an untrained population in a natural space when going through long periods of quarantine, until now.

Distress has generally been defined or divided between acute or chronic stress. The first one is a quick reaction to a situation that could develop into positive or negative emotions, though stress is typically related to negative affect. Distress is an interesting variable to analyze and study in an exceptional situation such as compulsory and obligatory lockdown, due to the possible post-traumatic stress disorders that could be developed. A prolonged situation of Distress can have a negative impact on the motivation needed to do activities that will act as a protective factor in the prevention of developing mood disorders. For example, high levels of stress influence a person’s life in many other aspects such as insomnia induced by high cortisol levels (Rodenbeck et al., 2002; Nandkar, 2020), furthermore it reduces the motivation to perform chores or responsibilities and includes other responses such as eating too much or not eating enough (Dua, 2019).

According to Watson (2000, p. 158) there exists a relation between stress or distress and feeling upset therefore, negative affect. But at the same time, people who tend to have negative emotions when experiencing high stress levels also tend to have negative emotions in low stress level situations. This could imply the importance of coping strategies when studying the duration or magnitude of stress. It is important to highlight that stress “*per se”* is not considered to be an emotion, but derives in negative or positive emotions; therefore, it is normally included within the negative affect dimension within mood, and its duration has been mainly studied in terms of hormones and neurotransmitters (Burke et al., 2005; Reeve, 2014, p. 75).

Stress has become the “disease” of the twenty-first century, according to the World Health Organization (Fink, 2016). It was initially a concept used to refer to neurobiological activations that take place with the presence of a stimulus that is considered harmful or dangerous (Smyth et al., 2013), and therefore “fight or flight” mechanisms begin due to the perception of a break up in the homeostasis of a person (Cannon, 1914). Nowadays, stress is not seen as “acute stress,” but rather seen as “chronic stress,” understood as feeling stress during a prolonged time frame (Senanayake and Arambepola, 2019). Traditionally, stress has been defined as the actions a person makes in order to react to a certain demand for change (Selye, 1965). Moreover, it is defined as a perceived feeling of too much demand (for example, work or chores) and not having enough resources to cope with the demands (Cherniss, 1980). It can also be defined as a threat being perceived by a person who feels incapable to cope with it (Biggs et al., 2017), based on Lazarus and Folkman’s transactional theory of stress and coping. In many cases, the concept of stress has been divided into “good” and “bad” stress, identifying eustress as “good stress” and Distress as “bad stress,” according to Selye (1976). However, Bienertova-Vasku et al. (2020) suggest that stress and Distress can be used indistinctively. As with any other emotion or feeling, stress can have the power to spill over and affect other people around you in a negative way. The term “adult coregulation” would also appear here. This means the power of “influencing each other’s moods and physiology” (p. 92) according to Saxbe and Repetti (2010).

One of the main obstacles or limitations in the papers and reviews consulted indicate that few studies follow subjects over time to observe the cyclical patterns of Distress, the study of its effect or forecasting the effect of distress, and how the different behaviors and activities that a person carries out influence Distress levels. In this sense, the studies found have generally focused on the population with mental health problems and some behaviors related to stress, such as tobacco consumption (Lawrence and Williams, 2016); subjects with medical problems, such as cancer survivors (Brinkman et al., 2013); or on specific situations of the subjects, such as marital disruption (Johnson and Wu, 2002); but not on general population cohorts, in a situation like the recently experienced lockdown, observing those daily life activities and behaviors that could be modulating experienced Distress.

The objective of applying pooled time series is to establish what effect Time has on the dependent variable (Distress) in different individuals. Pooled time series can detect the effect that the same variable has on itself through autoregressive models, where the independent variables (IVs) are past values of the same dependent variable (DV), and the different behaviors that people use to cope with Distress. In short, more quantitative studies are needed on the effect of a stressful phenomenon on the positive or negative mood of a person. In our case, we will study the Distress, as a variety of negative mood, produced by lockdown as a stressful event.

The use of autoregressive models is very useful since many physiological parameters and human behavior itself present cyclicity, regularity, and continuity. In addition, from a methodological perspective, cross-sectional models assume the serial independence of data, which does not occur in longitudinal studies since the longitudinal variable (DV) is correlated with past values of itself. Then, if longitudinal data were analyzed with cross-sectional models, Kmenta (1971, p. 283) demonstrated that the residuals will be autocorrelated, the parameters (*b*_0_, *b*_1_,…) are not biased, but the variances of the errors are underestimated. Therefore the variances and the standard errors of the parameters (that are in the denominator) also tend to be underestimated and, likewise, the values of the *t*, *z*, *F*, *R*^2^, and *b*_0_, *b*_1_… statistics are overestimated and not efficient, leading to type I errors (the assumption that a statistical effect exists, when in fact it does not) (Gujarati and Porter, 2013; Rosel et al., 2019). In addition, if we omit the values of the lagged variable, and this variable is part of the explanatory model of behavior, the coefficients obtained are biased and inconsistent, so the inferences drawn no longer have a substantive meaning (Gujarati and Porter, 2013; Draper and Smith, 2014).

This paper attempts to (a) assess the magnitude and duration of the influence of confinement on people’s Distress; (b) study the temporal sequence of stress, checking in what way and for how long the stress of any given day influences the subsequent Distress of a person; and (c) show how different activities carried out during the day (such as telecommuting, physical activity at home/building, have sexual activity and listening to music) influence in perceived Distress levels, using a daily survey. As we are working with temporal data, autoregressive models are more suitable to analyze cyclicality depending on the different daily activities that are carried out to face the lockdown. Our hypotheses have been established as follows:

a. Distress will have increased in each individual since the start of confinement, but then its impact has decreased over time.
b. Distress presents an autoregressive memory function, so that a high level of Distress during a given day will last for a period (up to 14 days).
c. There will be a set of variables and behaviors that will modulate the perceived Distress levels during the period of confinement. We hypothesized that gender and age affect the perceived Distress levels. The behaviors that have been considered are telecommuting, physical activity, sexual activity, and listening to music.
d. An interaction effect will be observed between gender (male/female) and Distress perceived “the day before” affecting men and women differently (*StD_*t*–1_⋅Gender*), as well as in variables and behaviors mentioned in “c.”

The intercept has been left as a Level 2 random variable (per participant). These hypotheses will be formalized in the data analysis from a regression equation, where the DV will be the daily Distress and the IV’s will be each of the variables indicated in the hypotheses in addition to the corresponding interactions of the variables, in order to verify its compliance or its empirical refutation, being the hypothetical equation:


$$S\,t\,D_{t\,k}=\left(b_{0}+b_{0\,k}\right)+\left[b_{1}\cdot S\,t\,D_{t-1}+b_{2}\cdot S\,t\,D_{t-1}\cdot G\,e\,n\,d\,e\,r\right]$$

$$+b_{3}\cdot S\,t\,D_{t-2}+b_{4}\cdot S\,t\,D_{t-3}+b_{5}\cdot S\,t\,D_{t-4}+b_{6}\cdot S\,t\,D_{t-5}$$

$$+b_{7}\cdot S\,t\,D_{t-6}+b_{8}\cdot S\,t\,D_{t-7}+b_{9}\cdot S\,t\,D_{t-8}+b_{10}\cdot S\,t\,D_{t-9}$$

$$+b_{11}\cdot StD_{t-10}+b_{12}\cdot StD_{t-14}+[b_{13}\cdot Telecommuting$$

$$-b_{14}\cdot Telecommuting\cdot Gender]-b{}_{15}\cdot SexualActivity$$

$$+\left[b_{16}\cdot L\,i\,s\,t\,e\,n\,t\,o\,m\,u\,s\,i\,c+b_{17}\cdot L\,i\,s\,t\,e\,n\,t\,o\,m\,u\,s\,i\,c\cdot G\,e\,n\,d\,e\,r\right]$$

$$+\left[b_{18}\cdot S\,p\,o\,r\,t+b_{19}\cdot S\,p\,o\,r\,t\cdot G\,e\,n\,d\,e\,r\right]+b_{20}\cdot G\,e\,n\,d\,e\,r$$

(1) $$-b_{21}\cdot A\,g\,e+\varepsilon_{t\,k}$$

In Equation 1 *b*_0k_ is the Level 2 intercept coefficient (participant), *k* refers to each individual in the sample; *b_0_, b_1,…_, b*_*j*_ are the coefficients of each variable, respectively; *StD* is the Stabilized Distress variable; and the main variables with their corresponding interactions have been placed between square brackets. The subscript “*t-1*” is the value of that same variable delayed 1 day, “*t-2*” delayed 2 days, and so on up to “*t-14*”.

## Materials and Methods

### Participants

The initial sample consisted of 319 participants recruited voluntarily through social media (web forums, WhatsApp, Twitter, and Facebook). Finally, 123 participants were selected from the total, because participants with less than 25 observations or non-consecutive registries were excluded. Participants were asked to respond to a socio-demographic survey first, where personal information and information about their physical activity and sleep quality pre-pandemic was requested. Several variables were collected in the sociodemographic questionnaire such as age, gender (male/female), education levels, marital status, number of people who were living with them during lockdown, and work status (essential/not essential) before the sanitary alert. From the fourth day after the start of the confinement in Spain (March 19th, 2020) until the de-escalation period (May 3rd, 2020), participants had to respond on the scales later described in the “Instruments and Variables” section. Due to the selection of participants carried out, Table 1 shows characteristics and basic differences of the group “participants” and “excluded.”

**TABLE 1:** Main descriptive statistics, including the contrast between excluded and selected subjects in the study.

		Excluded		Participants	
		N	%	N	%
Age [mean (*SD*)]		39^a^	11	43b	10
Gender	Woman	67^a^	27.70%	83^a^	34.30%
	Male	52^a^	21.50%	40^a^	16.50%
Studies	Primary/Basic	5^a^	2.00%	2^a^	0.80%
	Secondary (School/Vocational training)	36^a^	14.80%	26^a^	10.70%
	Higher (Advanced VT/University)	61^a^	25.00%	66^a^	27.00%
	Postgraduate (Master/PhD)	19^a^	7.80%	29^a^	11.90%
Marital status	Married/In union	59^a^	24.70%	73^a^	30.50%
	Separated/Divorced	10^a^	4.20%	15^a^	6.30%
	Single/Without a partner	47^a^	19.70%	35^b^	14.60%
COVID-19 diagnosed	Yes	2^a^	0.80%	2^a^	0.80%
	No	118^a^	48.60%	121^a^	49.80%
Shared dwelling	Living alone	11^a^	4.60%	22^a^	9.20%
	With one person	30^a^	12.60%	40^a^	16.70%
	With two persons	27^a^	11.30%	26^a^	10.90%
	With three or more	49^a^	20.50%	34^b^	14.20%
Physical health	Excellent	10^a^	4.30%	9^a^	3.80%
	Very good	28^a^	11.90%	35^a^	14.90%
	Good	56^a^	23.80%	60^a^	25.50%
	Not too bad	18^a^	7.70%	14^a^	6.00%
	Bad	1^a^	0.40%	4^a^	1.70%
Mental health	Excellent	12^a^	5.10%	10^a^	4.30%
	Very good	29^a^	12.30%	40^a^	17.00%
	Good	47^a^	20.00%	50^a^	21.30%
	Not too bad	21^a^	8.90%	19^a^	8.10%
	Bad	4^a^	1.70%	3^a^	1.30%
Sport practice	Yes	41^a^	18.14%	44^a^	19.47%
	No	64^a^	28.32%	77^a^	34.07%
Critical worker	Yes	43^a^	32.57%	40^a^	32.52%
	No	89^a^	67.42%	83^a^	67.48%
Smoker	Yes	46^a^	19.66%	32b	13.68%
	No. former smoker	24^a^	10.26%	33^a^	14.10%
	No. never	43^a^	18.38%	56^a^	23.93%

*Values in the same row and subtable not sharing the same subscript are significantly different at p < 0.05 in the two-sided test of equality for column proportions. Cells with no subscript are not included in the test. Tests assume equal variances. Tests are adjusted for all pairwise comparisons within a row of each innermost subtable using the Bonferroni correction.*

The final sample of 123 participants’ mean age was 42.80 (between 21 and 75 years old), with a standard deviation of 10.35 years. The sample included 40 men (32.5% of the total sample) and 83 women (67.5% of the total sample). Approximately half of the sample (*n* = 73, 59.3%) lived with a partner, whilst 40.7% (*n* = 50) were divorced or single. Up to 70% had university or postgraduate education (*n* = 95, 77.3%), 21.1% (*n* = 26) had secondary education and only 2 people (1.6%) had primary/basic education. Regarding sleep characteristics and physical activity pre-lockdown, we found that up to 70% had Fairly Good or Very Good sleep quality (*n* = 86, 74.8%) previous to the confinement vs. 25.2% (*n* = 29) who had Fairly Bad or Very Bad sleep quality. Regarding Physical Activity, 78.8% (*n* = 97) of the sample achieved the WHO recommendations, with 83.7% (*n* = 103) of the subjects performing intense or moderate physical activity before confinement. Of the total sample, 22 subjects (17.9%) lived alone during lockdown; the rest of the sample lived with their couple, children, parents, or other relatives, and/or pets. In Spain, critical workers (those necessary for the maintenance of basic social functions, health, security, social and economic well-being of citizens, or the efficient functioning of state institutions and public administrations) were allowed to go out to do their jobs (32.5% of the sample, *n* = 40), while non-essentials had to perform strict confinement and/or telecommute (67.5%, *n* = 83). Only 2 people (1.62%) were diagnosed positive with COVID-19 previously to the lockdown.

The 123 participants filled out a total of 4,924 daily records. However, when we introduce autoregressive variables of the same dependent variable up to a delay of 14 days, which causes the first 14 temporal data of each subject to be lost, the number of useful records is 3,132. No missing data have been estimated, and we have used the procedure of deleting the entire data line when there was any missing data, using the “LISTWISE” procedure in SPSS.

### Instruments and Variables

The main part of the daily record was comprised by the Adaptation of the Mood and Anxiety Symptom Questionnaire (MASQ-D30) Scale (Wardenaar et al., 2010) made by González and Ibáñez (2018) and also called MASQE-30, which comprises a total of 30 items designed to measure the three dimensions of the tripartite model of anxiety and depression: negative affect (NA), positive affect (PA), and somatic anxiety (SA) (Clark and Watson, 1991). According to Wardenaar et al. (2010) the three dimensions are called General Distress (equivalent to NA), Anhedonic Depression (equivalent to PA), and Anxious Arousal (equivalent to SA). The dependent variable in this study is Distress, which encompasses general symptoms of psychological Distress; that is, unpleasant feelings or emotions that can hinder people’s daily lives and affect the way one reacts to the people around them, especially when they feel overwhelmed. For this specific research, General Distress will be understood as the Generalized Least Squares (GLS) factor value, taken by maximum likelihood (ML), of Distress items obtained from each participant’s answers collected in the MASQE-30 daily register. The factorial variable Distress has a mean equal to 0, and a standard deviation of 1.225.

In addition, the daily records included questions about whether the participants had carried out work-related tasks in their homes using telecommuting that day (0 = not telecommuting and 1 = telecommuting that day); if they had practiced some kind of physical activity at home or in the building lasting more than 15 min (0 = had not practiced physical activity and 1 = had practiced physical activity that certain day); if during the day they had carried out sexual activity, regardless of whether it has been alone or accompanied (0 = did not have sexual activity and 1 = had sexual activity that day); if they had spent more than 15 min listening to music (0 = did not listen to music and 1 = listened to music that day). The effects of the levels of Distress perceived on previous days and some interactions between gender and those variables were considered (Yip and Tsang, 2007; Rosel et al., 2014).

### Procedure

The first step of this process was to create the online questionnaire using the platform interface of Qualtrics. Thanks to this application, we could automatically register the date and time at which the participant had completed the registration. In order to reduce the experimental mortality, a daily email was sent to the participants to remind them to complete the daily questionnaire. If the diary log was not answered, the next morning at 9:00 h an automatic reminder was sent to complete it.

All participants were recruited voluntarily through social media (web forums, WhatsApp, Twitter, and Facebook) along with a brief explanation of the study. The link to the socio-demographic survey was included. An email address was requested so that the participants could receive access to the daily diary. Once the first sociodemographic poll had been completed, the email containing the diary was sent toward the end of the day (20:30 h) to the participant’s email addresses. The diary was completed daily during the lockdown period. No specific conditions were required for the final sample to participate besides being adults (only adults over 18 years of age could participate in this study). All participants were living in Spain at the time of the lockdown and had access to the Internet.

The collected data were analyzed using the statistic procedure of lineal mixed-effects modeling in SPSS (2020). It is a multilevel model in which Level 1 is each measured variable, and Level 2 is each individual, that is, the respective measurements are nested within each individual (Gill, 2005; Goldstein, 2010). The model estimation and factor analysis were done with ML extraction. The general regression equation can be decomposed into the corresponding equation for each group (male/female), and because the variable gender has two levels (groups) we can obtain a different equation for males and females that represents the perceived Distress (Yip and Tsang, 2007; Rosel et al., 2014; Hayes and Montoya, 2017).

To measure Distress memory, first the configuration of the data has been changed from individual per line to data-day per line (Singer and Willett, 2003), and then the stabilized Distress variable has been delayed up to 14 days in each participant, with the precautions to be taken in the pooled time series 256 models (Sayrs, 1989; Andreß et al., 2013; Rosel et al., 2019). Furthermore, when time series data are used, it is assumed that serial correlation exists, which means that a person’s mean Distress level of that day will affect their mean Distress level of the next day/s.

**FIGURE 1:**
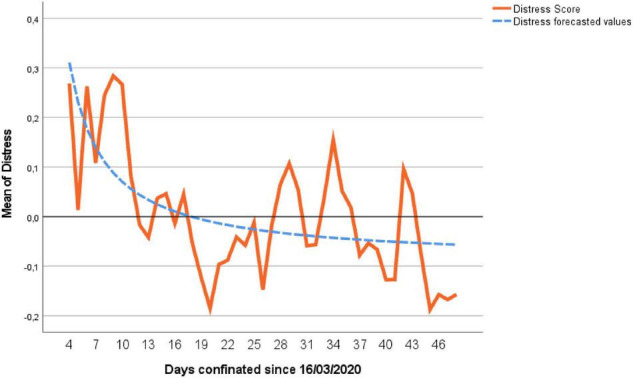
Mean values of Distress for all the sample and predicted values obtained by Equation 2.


According to Box and Jenkins (1976) recommendations for time series analysis, when a time series doesn’t keep stability in its mean, differentiation would be recommended. If we observe Figure 1, the mean of Distress is higher at the beginning of confinement and tends to decrease over time and slowly stabilize. However, if the differentiation procedure is followed, the dynamic properties of the time series are lost in the long term (Huckfeldt et al., 1982; Engle and Granger, 1987). The decreasing Distress can be formalized through the reciprocal function *(1/t)*, where “t” is the number of days elapsed since confinement. Function *1/t* has been taken as the stabilization variable of the series due to its simplicity and because it presents great flexibility when adjusting curves with an initial rapid decrease (or increase), later presenting an asymptotic stable almost horizontal trend. The following regression has been made:

(2) $$D\,i\,s\,t\,r\,e\,s\,s_{t}=b_{0}+b_{1}\,\left(\frac{1}{t}\right)+\varepsilon_{t}$$

Where ε_t_ is the part of the Distress factor not explained by the reciprocal function in Equation 1, which is now stationary in the mean. The values of ε_t_ will be called stabilized Distress. The stabilized (ε_t_) model is a multilevel model as each individual represents a different level. Equation 1 has not been calculated in a multilevel way to allow general Equation 3 to capture the possible multilevel relationship between the intercept and the DV. The mentioned model includes an analysis of principle fixed effects of Distress, the effect of previous days (lags), and the effect of different variables considered in the design (telecommuting, housework, physical activity, sexual activity, listening to music). Interactions between gender and the variables mentioned before were considered, and the Level 2 of the intercept, each individual.

### Ethical Standards

The authors assert that all procedures contributing to this work comply with the ethical standards of the relevant national and institutional committees on human experimentation and with the Helsinki Declaration of 1975, as revised in 2008. The Deontological Commission of the Jaume I University has issued a favorable report on the project with file number CD / 24/2020: “Mood evolution during confinement due to COVID-19 in Spain,” considering that it complies with the deontological regulations required.

## Results

A significance level has been taken for the results of 5% (α = 0.05). The following consistency statistics were observed: Cronbach’s α = 0.983; Intraclass Correlation (ICC) for single measures = 0.568 (47, 2,068), *p* < 0.001; ICC for average measures = 0.983 (47, 2,068), *p* < 0.001. The statistics Partial Eta Squared (η^2^), Non-centrality Parameter (δ), and Observed Power (1-β) are also indicated. The Distress score in function of the variable “1/time” (Equation 1) was statistically significant (*F* = 16.270; *df*: 1, 4,854; *p* < 0.001; η*^2^* = 0.003; δ = 16.270; *1*-β = 0.981), obtaining the coefficients:


(3) $$D\,i\,s\,t\,r\,e\,s\,s_{t}=-0.090+1.604\,\left(\frac{1}{t}\right)+\varepsilon_{t}$$

We can see in Figure 1 how the trend of the series is well represented in the predicted values obtained in Equation 3, superimposing these predicted values to those obtained empirically by the sample. One of the objectives of this study was to assess the magnitude and duration of the influence of confinement on people’s Distress. Although the first 3 days from the start of the lockdown could not be measured, it is observed that the maximum Distress took place during the first 7 days measured (days 4–10 after lockdown), as the mean of these days is equal to 0.211 (*SD* = 1.355). Taking as a reference the last 10 days of confinement, the data closest to the possible “normality” (Figure 1) because they are the ones that will most resemble in level and variability to the data of a normal period, the sample has had a Distress *M* = −0.089, and *SD* = 1.117. Because the data have two levels, a comparison of means (Student’s *t*-test) was made with bootstrap with the criterion of stratification per participant (level 2) with 1,000 subsamples (Pons, 2007; Wu and Thompson, 2020), giving a value of *p(t)* = 0.001, bias = −0.001, Cohen’s *d* = −0.247, 95% CI [(−0.414) to (−0.200)]. Therefore, the difference in the level of anxiety from the beginning of the pandemic to that of the end is significant. The bootstrap system does not give a value of *t* but, under normal conditions and *p(t)* = 0.001, corresponds a t* = 3.301, which indicates that at the beginning of the pandemic our sample suffered very high levels of Distress, approximately 3.301 scores typified in relation to the distress suffered at the end. As we have a very large sample, the values of *t* are equivalent to the values of *z*.

**FIGURE 2:**
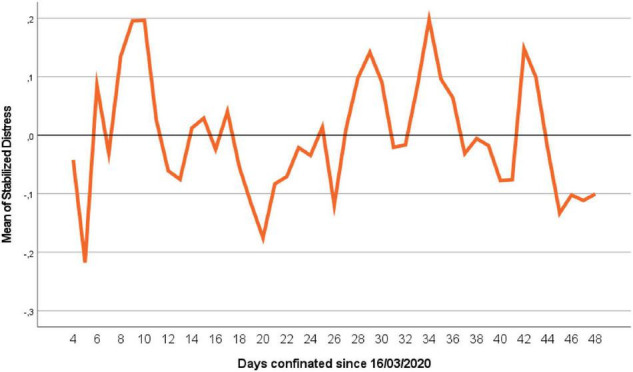
Stabilized values of Distress for all the samples.

The values of these forecast errors are the part of the Distress factor not explained by the reciprocal function of “*t*” in Equation 1, so these values of ε_t_ will be called Stabilized Distress (ε_t_). Then, considering only the stabilized Distress, we can represent it graphically as in Figure 2, which is now already stationary on average, and it is what we will use as DV from now on to forecast the stabilized Distress in Equation 1.

An exploratory analysis of stabilized Distress has been made, and it has been verified that there are quite a few significant lags in the ACF and PACF, in addition to the Box-Ljung statistic having a value for 14 lags of 22785.740, *df*: 14, *p* < 0.001. So, the data of the series are not “white noise,” presenting serial dependence, and must be modeled using time series with the variables presented in the hypothesis. Given that our hypothesis on the lags is autoregressive, so that more recent lags will influence more than the older ones, the non-significant intermediate lags have been maintained as indicated by Box et al. (2016); delays 7 and 14 are maintained, since we see that the behavior presents a seasonality of 7 days. We have suppressed the variables Lag 1 stabilized Distress Gender (*b* = 0.002, *SE* = 0.023, *p* = 0.934), Age (*b* = −0.001, *SE* = 0.001, *p* = 0.174), and the Listening interaction music Gender (*b* = 0.101, *SE* = 0.054, *p* = 0.062) because they are not statistically significant. The final stabilized model can be observed in Table 2.

**TABLE 2:** Estimates of fixed effects included in the final stabilized model of distress during lockdown.

Parameter	Estimate	Std. Error	*t*	*p*	η*2*	δ	1-*β*
Intercept	0.040	0.030	1.312	0.190	0.001	1.303	0.256
Lag 1 stabilized distress	0.313	0.018	17.607	0.000	0.090	17.579	1.000
Lag 2 stabilized distress	0.106	0.018	5.777	0.000	0.011	5.816	1.000
Lag 3 stabilized distress	0.031	0.018	1.656	0.098	0.001	1.677	0.388
Lag 4 stabilized distress	0.073	0.018	3.992	0.000	0.005	3.952	0.977
Lag 5 stabilized distress	0.100	0.018	5.490	0.000	0.009	5.456	1.000
Lag 6 stabilized distress	0.080	0.018	4.432	0.000	0.006	4.377	0.992
Lag 7 stabilized distress	0.064	0.018	3.488	0.000	0.004	3.495	0.938
Lag 8 stabilized distress	0.019	0.018	1.070	0.285	0.000	1.097	0.195
Lag 9 stabilized distress	0.022	0.018	1.227	0.220	0.000	1.217	0.229
Lag 10 stabilized distress	0.051	0.017	2.960	0.003	0.003	2.948	0.838
Lag 14 stabilized distress	0.049	0.015	3.276	0.001	0.003	3.225	0.897
Telecommuting	0.088	0.046	1.895	0.058	0.001	1.884	0.469
Physical activity	0.013	0.050	0.251	0.802	0.000	0.248	0.057
Sexual activity	−0.127	0.039	−3.236	0.001	0.003	3.222	0.896
Listen to music	−0.092	0.025	−3.712	0.000	0.004	3.683	0.957
Gender	0.079	0.034	2.309	0.021	0.002	2.283	0.627
Telecommuting ⋅ Gender	−0.156	0.056	−2.786	0.005	0.002	2.766	0.790
Physical activity ⋅ Gender	−0.113	0.060	−1.903	0.057	0.001	1.888	0.471

*Dependent variable: Stabilized distress. η^2^, Partial Eta Squared; δ, Non-cent. Parameter; 1-β: Observed Power.*

There is no significant Level 2 inter-subject differences (intercept, Var[*b*_0k_] = 0.000), which means that all subjects present the same intercept of Distress but not all individuals present the same level in the values obtained in the delays and in the variables included in the equation.

In order to test the significance of the total fit of the equation in Table 2, the value of its −2 logarithm of the likelihood (−2LL) is compared with its respective parameters (−2LL = 6396.565; parameters: 21), with that of the null model, only with the intercept of the series (−2LL_0_ = 15737.478; parameters: 2), being the increment of values: Δ(−2LL) = 9340.913, and Δ(*df*) = 19, which follows a chi-squared distribution, so the fit of the model of the equation in Table 2 has a *p* < 0.001, the overall fit being significant. The *R*^2^-value is 0.663 (*p* < 0.001), so approximately 66% of the variance of Distress is explained by the equation resulting from the variables included in Table 2.

Table 2 shows that the variables related to physical activity (Physical Activity and Physical Activity Gender) are not significant, but when we have removed them from the final equation, their Akaike information criteria (AIC) has passed from 6396.565 with these 2 variables (model in Table 2) to 6406,272, so apparently the model with physical activity is better than without it. We have verified if this improvement is statistically significant by comparing their respective −2LL, where the −2LL = 6392.467 for the model without Physical Activity and Physical Activity Gender, so Δ(−2LL) = 6406.272–6396.565 = 9.707, Δ(*df*) = 21–19 = 2, *p* = 0.008, so the difference is significant in favor of the model with the physical activity variables in Table 2. In other words, although the Physical Activity and Physical Activity Gender variables are not significant separately, when their overall probability is calculated, it is significant, so we leave them in the final Equation of Table 2.

**FIGURE 3:**
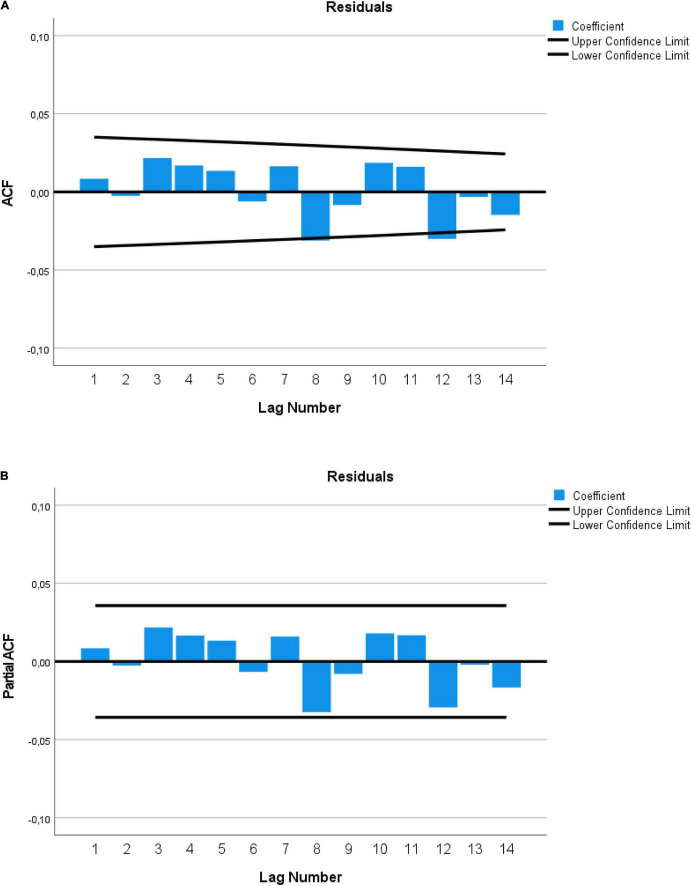
**(A)** ACF and **(B)** PACF graphics for residuals of equation of Table 2 of Distress.

Finally, the study of the model residuals is carried out to determine if they constitute white noise. As we can see in the ACF and PACF (Figures 3A,B), we can determine that the time series for Stabilized Distress is correctly modelized, including the first 10 lags and the 14th lag, as well as a group of variables that contribute to the explanation of the Distress score (Box-Ljung statistic for 14 lags of 19.808, *df*: 14, *p* = 0.136). In Figure 2A, delays 8 and 12 are at the limit of significance, but bear in mind that for a delay to be significant it must be in its ACF and its PACF, and since they are not significant delays 8 and 12 of Figure 2B, nor the result of the Box-Ljung test, the residuals constitute a “white noise.” In Box-Jenkins terms, the model has a memory of 10 simple days plus a seasonality of 7 days over 2 weeks, that is, it is an ARIMA(10,0,0) (2,0,0)_7_ model. This check is very important from a statistical perspective, because it indicates that the coefficients obtained are not biased and their standard errors are consistent, avoiding type I errors (Kmenta, 1971). That is, the effects obtained are significantly so in reality. In summary, since the overall fit of the model is significant, and the residuals are “white noise,” we accept the model from Table 2.

Turning now to the next part of the results of the model proposed shown in Table 2, interactions between gender and some variables can be highlighted. Developing Stabilized Distress (*ε_t*k*_*), according to Table 2:


$$\varepsilon_{t\,k}=0.040+0.313\,\text{⋅}\,S\,t\,D_{t-1}+0.106\,\text{⋅}\,S\,t\,D_{t-2}+0.031\,\text{⋅}\,S\,t\,D_{t-3}$$

$$+0.073\,\text{⋅}\,S\,t\,D_{t-4}+0.100\,\text{⋅}\,S\,t\,D_{t-5}+0.080\,\text{⋅}\,S\,t\,D_{t-6}$$

$$+0.064\,\text{⋅}\,S\,t\,D_{t-7}+0.019\,\text{⋅}\,S\,t\,D_{t-8}+0.022\,\text{⋅}\,S\,t\,D_{t-9}$$

$$+0.051\text{⋅}StD_{t-10}+0.049\text{⋅}StD_{t-14}+[0.088\text{⋅}Telecommuting$$

-0.156⋅Telecommuting⋅Gender]-0.127⋅SexualActivity

-0.092⋅Listentomusic++[0.013⋅PhysicalActivity

(4) $$-0.113\text{⋅}PhysicalActivity\text{⋅}Gender]+0.079\text{⋅}Gender+\varepsilon {}_{t\,k}{}^{{}^{\prime }}$$

In Table 2 it can be seen that the delays 3, 8, and 9 of the Stabilized Distress variable are not significant, but they have been included due to the fact that the subsequent delays are significant, that is, delay 4 and delay 10 (Box et al., 2016). Note how all the autoregressive coefficients are positive, which is consistent with the expected behavior of any individual, so that if Distress increases on any given day, stress will tend to increase in subsequent days.

Previously it has been explained why Physical Activity and Physical Activity Gender have been included, but it can also be seen that there is a simple variable, Telecommuting, that is not significant and has been kept in the equation. This is because it is part of a significant interaction (Telecommuting Gender), so it must be maintained due to the principle of “nesting” in the interaction of variables. An important aspect to take into account is that when the interaction of gender with another variable is significant, the coefficient of that variable is different for men and women (Hayes and Montoya, 2017). Thus, the interaction of the Physical Activity and Telecommuting variables by gender indicates that both variables affect men differently than women in terms of their effect on Distress. Specifically, due to its negative sign and the Gender values being 0 for male and 1 for female, we can affirm that both variables reduce the perceived Distress in women.

Considering that in Equation 3, *ε_t*k*_* is a part of Distress indicated in Equation 2, we can substitute *ε_t*k*_* from Equation 4 in Equation 3:


$$D\,i\,s\,t\,r\,e\,s\,s_{t\,k}=-0.090+1.604\,\left(\frac{1}{t}\right)+\left\{\varepsilon_{t}\right\}$$

$$=-0.090+1.604\left(1/t\right)+\{0.040+0.313\cdot StD_{t-1}$$

$$+0.106\cdot S\,t\,D_{t-2}+0.031\cdot S\,t\,D_{t-3}+0.073\cdot S\,t\,D_{t-4}$$

$$+0.100\cdot S\,t\,D_{t-5}+0.080\cdot S\,t\,D_{t-6}+0.064\cdot S\,t\,D_{t-7}$$

$$+0.019\cdot S\,t\,D_{t-8}+0.022\cdot S\,t\,D_{t-9}+0.051\cdot S\,t\,D_{t-10}$$

$$+0.049\cdot StD_{t-14}+[0.088\cdot Telecommuting$$

-0.156⋅Telecommuting⋅Gender]-0.127⋅SexualActivity

-0.092⋅Listentomusic+[0.013⋅PhysicalActivity

(5) -0.113⋅Sport⋅PhysicalActivity]+0.079⋅Gender+εt⁢k}′

The final Equation 5 can be developed in two different equations for men and for women. Therefore, because it has been coded with the value of “0” for men and “1” for women, Equation 2 is substituted and simplified for each gender, and each corresponding interaction is replaced by its result, as follows:


$$D\,i\,s\,t\,r\,e\,s\,s_{M\,a\,l\,e_{t\,k}}=0.050+1.604\,\text{⋅}\,\left(\frac{1}{t}\right)+0.313\cdot S\,t\,D_{t-1}$$

$$+0.106\cdot S\,t\,D_{t-2}+0.031\cdot S\,t\,D_{t-3}+0.073\cdot S\,t\,D_{t-4}$$

$$+0.100\cdot S\,t\,D_{t-5}+0.080\cdot S\,t\,D_{t-6}+0.064\cdot S\,t\,D_{t-7}$$

$$+0.019\cdot S\,t\,D_{t-8}+0.022\cdot S\,t\,D_{t-9}+0.051\cdot S\,t\,D_{t-10}$$

$$+0.049\cdot S\,t\,D_{t-14}+0.088\cdot T\,e\,l\,e\,c\,o\,m\,m\,u\,t\,i\,n\,g$$

-0.127⋅S⁢e⁢x⁢u⁢a⁢l⁢A⁢c⁢t⁢i⁢v⁢i⁢t⁢y-0.092⋅L⁢i⁢s⁢t⁢e⁢n⁢t⁢o⁢m⁢u⁢s⁢i⁢c

(6) +0.013⋅P⁢h⁢y⁢s⁢i⁢c⁢a⁢l⁢A⁢c⁢t⁢i⁢v⁢i⁢t⁢y+εt⁢k′

$$D\,i\,s\,t\,r\,e\,s\,s_{F\,e\,m_{t\,k}}=0\,.029+1.604\,\text{⋅}\,\left(\frac{1}{t}\right)+0.313\cdot S\,t\,D_{t-1}$$

$$+0.106\cdot S\,t\,D_{t-2}+0.031\cdot S\,t\,D_{t-3}+0.073\cdot S\,t\,D_{t-4}$$

$$+0.100\cdot S\,t\,D_{t-5}+0.080\cdot S\,t\,D_{t-6}+0.064\cdot S\,t\,D_{t-7}$$

$$+0.019\cdot S\,t\,D_{t-8}+0.022\cdot S\,t\,D_{t-9}+0.051\cdot S\,t\,D_{t-10}$$

$$+0.049\cdot S\,t\,D_{t-14}-0.068\cdot T\,e\,l\,e\,c\,o\,m\,m\,u\,t\,i\,n\,g$$

-0.127⋅S⁢e⁢x⁢u⁢a⁢l⁢A⁢c⁢t⁢i⁢v⁢i⁢t⁢y-0.092⋅L⁢i⁢s⁢t⁢e⁢n⁢t⁢o⁢m⁢u⁢s⁢i⁢c

(7) -0.100⋅P⁢h⁢y⁢s⁢i⁢c⁢a⁢l⁢A⁢c⁢t⁢i⁢v⁢i⁢t⁢y+εt⁢k′

We can observe different values of the intercept for men (0.050) and for women (0.029). This is because in men (Equation 6) it is the result of the sum of the intercepts of Equation 3 (−0.090) and Equation 4 (0.040); while in women (Equation 7) it is the previous result plus the value of 0.079⋅*Gender* = 0.079, being the result of 0.090 + 0.040 + 0.079 = 0.029. In the same Equations 6 and 7 the effect of 1/*t* is significant (*b* = 1.604); this indicates that when the time from confinement increases, its effect on Distress decreases until it approaches the value of −0.090 (Distress baseline determined by the intercept of Equation 3). Initial values of Stress are very high, but they decrease gradually, rapidly in the beginning, and more slowly from day 30 (Figure 1). This can be seen with an example: in the case of keeping all the other variables of the Equation 5 constant, on the first day of confinement (*t* = 1) the increase in Distress will be 1.604 points; on the 15th day of confinement (*t* = 15), the increase in Distress due time was: 1.604⋅(1/15) = 0.107; on the 30th day of confinement (*t* = 30), the increase in Distress due time was: 1.604⋅(1/30) = 0.053; on the last day of lockdown, the increase in Distress was 0.033 [1.604⋅(1/30)] verifying that when *t* increases, *1*/*t* approaches the value 0.

The proposed model has significant delays up to 14 days, which indicates that during the lockdown the “memory” of Distress has been 2 weeks (Box and Jenkins, 1976). The term 0.313⋅*StD*_*t*–1_ in the equation means that if 1 day any person’s Distress increases by one unit, the next day their Distress increases by 0.313 units. In the same way,.106⋅*StD*_*t*–2_ indicates that for each unit of increase in Distress, after 2 days Distress will increase by 0.106 units, and so on until the 14th delay was reached, when term 0.049⋅*StD*_*t*–14_ indicates that an increase in one unit of Distress would produce an increase of 0.049 units of Distress after 14 days.

Regarding the term [0.088⋅Telecommuting −0.156⋅Telecommuting⋅Gender] of Equation 5, as the Telecommuting⋅Gender interaction was significant (*p* = 0.005), the simple variables telecommuting and gender have also been left, resulting for men: 0.088⋅Telecommuting −0.156⋅Telecommuting⋅0=0.088⋅Telecommuting; and for women: 0.088⋅Telecommuting −0.156⋅Telecommuting⋅ 1 = −0.068⋅Telecommuting (Equations 6 and 7). Therefore, the coefficients are different for men and women: in men, the fact of teleworking increases Distress by 0.088 units, while in women it decreases by 0.068 units. The gender coefficient value (0.079) is not included because it has already been incorporated before, and it must be included only once in Equation 7.

Regarding the variable “Sexual Activity” (*b* = −0.127, *p* < 0.001), the practice of sexual activity reduces distress for a day by 0.127 units, and this occurs in both men and women.

We also observe in Table 2 that the Physical Activity⋅Gender interaction has been no significant (*b* = −0.113, *p* = 0.057), being the value of its interaction (0.013⋅Physical Activity −0.113⋅Physical Activity⋅Gender), plus the effect of gender, which has already been included (Equations 6 and 7). This would indicate that Physical Activity practice affects distress differently depending on gender, although the practice of physical activity as a principal variable is not significant (*b* = 0.013, *p* = 0.802), as seen in Table 2. Therefore, in the case of men: 0.013⋅Physical Activity −0.113⋅Physical Activity⋅0 = 0.013⋅Physical Activity, which would indicate that the practice of physical activity increases distress in men by 0.013 points (although this value in itself is not significant, it is maintained because together with Physical Activity it is significant). On the other hand, in women, 0.013⋅Physical Activity −0.113⋅Physical Activity⋅1 = −0.100, which means that the practice of physical activity reduces distress in women by 0.100, with significant differences between men and women.

Finally, the observed power (*1*-β) of the variables has been calculated, the lowest being that of the Physical Activity variable (*1*-β = 0.057), although the interaction of Physical Activity and Gender is higher (β = 0.471), and its coefficient is not significant (*b* = −0.113, *p* = 0.057). Possibly it would be convenient to expand the sample, but if we take into account that both variables together (*p* = 0.008) then the sample size is adequate. That is, the power of the Physical Activity variable is associated with that of the Gender variable and the interaction of both variables. The highest power has been achieved in the variable *StD*_*t*–1_ (β = 1.000), together with 1/t (β = 0.981), so that the temporary variables have great power, making our sample sufficiently representative for robust effect parameters.

## Discussion

Distress, as well as other variables that can have a negative impact on a person’s well-being, have been studied in other countries, and results tend to agree on the importance of focusing on the population’s psychological and emotional well-being and thus highlighting mental health as a protection factor in future lockdown situations that could take place. Moreover, some variables that can act as protective factors are importantly studied in order to prevent negative consequences in extreme situations such as compulsory lockdown or any other situations where adaptation is necessary, in order to increase the efficiency of coping strategies and resilience. One of the main goals of the current study was to analyze and explore the effects of daily activities on Distress levels and fluctuations during the COVID-19 pandemic lockdown in Spain. Taken together, these findings suggest the importance for public health to apply measures on wellbeing.

These findings broadly support the work of other studies in Distress that link its gradual reduction with the maintenance of the situation over time. In this sense, we can observe that in the first days of the confinement a high level of Distress is observed, decreasing rapidly in the following days, and more slowly in the final periods of the lockdown (Figure 1). As an interesting detail, it should be noted that on days 20, 34, and 49 from the beginning of the closure, extensions of the state of alarm were approved in the council of ministers, although the media gave the news of the extension proposals 4–5 days before. These approvals and notifications coincide closely with abrupt increases in the level of Distress of the subjects studied. These increases in distress in the face of the extensions of the confinement are logical, since the perceived insecurity about the development of the pandemic, the economic and labor consequences both for oneself and for their acquaintances and relatives, the perception of the risk of contagion and serious course of the disease, and the length of the confinement itself are the main causes of perception of insecurity and distress.

An important finding to take into account is that if the Distress of our sample is taken during the first 7 days of our investigation (from the fourth day of confinement), and it is compared with the mean Distress during the last 10 days of confinement (with a lower mean Distress, and supposedly more similar to Distress under normal conditions), then the *z*-value (standardized score) increased by 3.301 points (*p* = 0.001), which indicates that the participants endured a very high level of Distress. If this comparison were made with a sample during a normal unconfined phase, this value of *z* would probably increase.

Other significant finding to emerge from this study is that during the lockdown, the “memory” of Distress has been 2 weeks, resulting in a significant inertia within each person where no differences are observed between people in the process generation of Distress, because the Level 2 coefficient is not significant. Overall, these results indicate that levels of Distress also depend on the activities that a person did during lockdown. In addition, interaction exists between gender and some behavioral variables. This means that a woman who did some of the activities mentioned during the day would show a different level of Distress compared to a person who did not do these activities, making the differences statistically significant.

Thus, there are different behavior variables like Telecommuting, which increases Distress in men by 0.088 points, but decreases 0.068 in women; or Physical Activity, which increases Distress 0.013 points in men but decreases Distress by 0.100 points in women. Comparing the results, women were generally positively influenced by telecommuting and practicing physical activity more than men. Having sexual activity influences positively both genders.

Significant statistical differences were found when considering Distress as a function of lag 1, lag 2, lag 4 to lag 7, lag 10, and lag 14. The results of this investigation show that, during lockdown, people’s Distress levels from previous days influenced the Distress levels of that day, which meant that what happened today will affect your Distress levels up to 14 days later, but the effect is higher during the two following days, with the biggest values of lag coefficients. Furthermore, females had, overall, higher Distress levels during the COVID lockdown. A total of 56.25% (*n* = 72) of the participants telecommuted, and Distress levels in men were higher compared to those participants who did not telecommute, but in women this activity decreased Distress. Having sexual activity reduced Distress levels in general, with no effect differences being male or female. Furthermore, music is a protective factor for both men and women in keeping Distress levels lower. Age did not influence the level of Distress experienced by the sample. Focusing on levels of Distress in women as compared to men, doing daily physical activity reduced their daily perceived levels of Distress, proving the importance for women of doing daily activities to reduce Distress levels. This study proves that gender differences must be considered in order to analyze correctly analyze lockdown data.

It is interesting to note that the general intercept *b_0_*, was estimated as a fixed effect and as a random effect (individual effect Level 2 variable) but this last one did not show significant statistical differences. This discrepancy could be attributed to a large time memory of 14 days, with 11 autoregressive terms, from *StD*_*t*–1_ to *StD*_*t*–10_ including *StD*_*t*–14._ These 11 terms reflect every person’s distress level, thus high levels of *StD*_*t*–1_ to *StD*_*t*–14_ will forecast high levels of the dependent variable Distress, not being necessary to reflect it in a Level 2 intercept variability. Therefore, the fact that our individuals have a common intercept does not mean that they also have the same level of Distress, since their daily level is conditioned by the levels of the previous 14 days.

It might seem that the main weakness of this study was the number of observations registered per participant when considering a time series. According to Box et al. (2016, p. 31) “*to obtain a useful estimate of the autocorrelation function in practice, we would typically need at least 50 observations*” but having used a pooled time series or panel data system, the statistical power is much higher (Baltagi, 2005). However, our research findings show that the time variables had higher statistical power than cross-sectional variables, and we consider that our sample has sufficient statistical power to assume that it includes a sufficient number of participants. The strict confinement in Spain lasted 48 days, then, the de-escalation period began, allowing certain confinement relief measures at different stages. In addition, a daily record is difficult for the participants to follow, which means that there were omissions in the completion of several consecutive days, in approximately 2/3 of the subjects or that they completed less than 20 days, proceeding to their elimination of the study sample. Future research could usefully consider in-depth analysis to study the temporary process in order to analyze the trajectories and changes of the main variable over time. It is probable that the autoregressive effects and the effects of the significant variables in other situations will be very similar to those found during confinement. Probably, the behavior of individuals during a period of “normality” without a pandemic will show lower levels of distress and less variability.

The sample of participants used has been made up of volunteers and unpaid people, so there are variables that are not sampled in a “balanced” manner (gender, age, telecommuting, if they practice regular physical activity,…) and, therefore, it could be argued that it is not sociologically representative. On the other hand, it has the advantage that it presents ecological representativeness, since it is assumed that the process of distress change has been very similar in our sample and in the general population. In addition, without forgetting the sample representativeness, special attention has to be paid to the representativeness of the process studied in the research carried out (Brunswik, 1956; Bordalo et al., 2021). More studies are needed to see if the psychological effects of Distress during the pandemic have been short-lived or if they have a longer-term effect.

The high levels of Distress suffered by our sample suggest that some type of support system should be considered for successive occasions. At the end of the confinement, it suggests that the population was subjected to a psychological pressure far above what is clinically normal. We hope that these high levels of Distress suffered at the beginning of the pandemic will not leave pathological traces in individuals. Faced with future confinement situations, it would be necessary to have detection systems for people with a high level of Distress and have prepared intervention protocols to intervene individually in high-risk people (personal or social), and globally (through campaigns of awareness, establishment of routines, schedules, physical activity, hobbies, use of social networks and teleconferences, etc.) in the population.

In addition, computerized registration systems allow almost continuous data collection, as in the present investigation. Different variables have been collected over multiple days belonging to different individuals, which requires data analysis models appropriate to the system used, so it is expected that training in data-intensive analysis models using pooled time series and other techniques (multilevel analysis, resampling,…) will increase (Nusser et al., 2006; Bolger and Laurenceau, 2013; Walls, 2013; Rosel et al., 2020). So, future studies on the temporal processes of Distress should be carried out to check if its memory and the influential independent variables on Distress are similar during confinement and under non-confinement conditions, as in the “de-escalation” of lockdown limitations, and in periods of normality.

## Data Availability Statement

The original contributions presented in the study are included in the article/supplementary material, further inquiries can be directed to the corresponding author/s.

## Ethics Statement

The studies involving human participants were reviewed and approved by the Deontological Commission of the Jaume I University with file number CD/24/2020: “Mood evolution during confinement due to COVID-19 in Spain”. The patients/participants provided their written informed consent to participate in this study.

## Author Contributions

PF-A: writing—original draft, data curation, formal analysis, and writing—review and editing. EF: supervision and writing—review and editing. JR: supervision, validation, formal analysis, and writing—review and editing. AB-L: supervision, validation, and writing—review and editing. FM: conceptualization, writing—original draft, formal analysis, and writing—review and editing. All authors contributed to the article and approved the submitted version.

## Conflict of Interest

The authors declare that the research was conducted in the absence of any commercial or financial relationships that could be construed as a potential conflict of interest.

## Publisher’s Note

All claims expressed in this article are solely those of the authors and do not necessarily represent those of their affiliated organizations, or those of the publisher, the editors and the reviewers. Any product that may be evaluated in this article, or claim that may be made by its manufacturer, is not guaranteed or endorsed by the publisher.
